# Prevention, Detection and Response to incidences of substandard and falsified medical products in the Member States of the Southern African Development Community

**DOI:** 10.1186/s40545-020-00257-9

**Published:** 2020-10-20

**Authors:** Stanislav Kniazkov, Sakhile Dube-Mwedzi, Jean-Baptiste Nikiema

**Affiliations:** 1 WHO Regional Office for Africa, Medicines, Infrastructure and Medical Equipment Unit, Brazzaville, Republic of Congo; 2 SADC Medicines Regulatory Harmonization Project, Harare, Zimbabwe

**Keywords:** Substandard and falsified, Prevention, Detection and Response (PDR), Global surveillance alerts

## Abstract

**Background:**

Medical products are an integral and pivotal part of health care delivery. They need to be available, affordable and quality-assured. The SADC region is prone to threats arising from the availability and use of substandard and falsified (SF) medical products. This is something that needs to be actively addressed.

**Method:**

A survey, constructed around four themes, was carried out between September 2018 and January 2019. The National Medicines Regulatory Authorities (NMRAs) of the 16 Member States within the SADC region were asked to respond to the survey questionnaire. The objective was to map existing fameworks, mechanisms and approaches to prevention, detection and response (PDR) to SF medical products.

**Results:**

Responses were received from twelve out of the sixteen NMRAs. Only three of the twelve respondents had included elements for PDR for SF medical products in their national medicine policies. Regardless of the status in terms of policies, legislation is however in place for the majority of NMRAs. The mandate for regular sampling, an important detection mechanism, was enshrined in the legislation of nine of the twelve respondents. In terms of response mechanisms, six of the respondents had both inter-agency and intra-agency co-ordination for responding to SF products.

**Conclusion:**

Though findings point to some deficiencies in terms of policies and implementation plans, the majority of countries have the mandate and legislation to deal with substandard and falsified medical products. Effective enforcement requires more investments into human resources, infrastructure, stakeholder coordination and public outreach. WHO has an important source of actionable information about incidience of substandard and falsified medical products. It needs to be leveraged to improve outreach to stakeholders and to raise awareness about SF problem and mechanisms available to address it. The extent, to which mechanisms and procedures are in place, varies. Some elements of the desired approach exist in the region; however, they will benefit from targeted strengthening to ensure a holistic approach across 12 action areas recommended by WHO.

## Background

Access to essential medicines and health products holds significant importance in addressing health problems [1]. However, it can be gravely undermined by the existence of substandard and falsified (SF)^1^ medical products. They are a public health threat and an impediment to the universal access to medical products. This is acknowledged by Funestrand et al., who also highlight that regulations and penalties to deter their trade differ globally [2]. SF medical products harm patients and reduce confidence in medical products, health professionals and health system [3]. According to WHO, 1 in 10 medical products in developing countries is substandard or falsified [4].

### The problem of substandard and falsified medical products

**Fig. 1:**
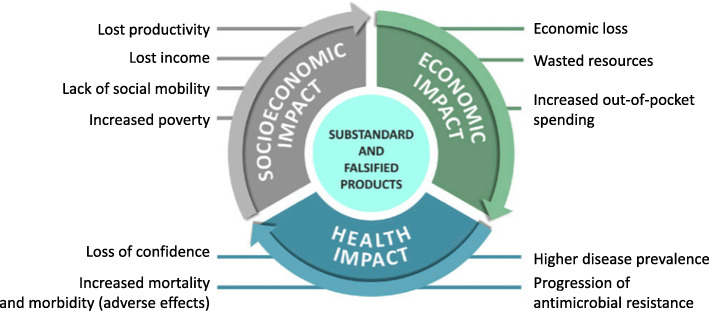
Impact of substandard and falsified medical products. *Source: WHO Geneva. 2017*

The WHO study on the public health and socioeconomic impact of SF medical products articulates the problem and impact of SF products. The study establishes that these products threaten the health of those who take them, as they may be of poor quality, unsafe or ineffective. The study highlights the impact of SF products used in the first-line treatment of uncomplicated malaria: incremental deaths in sub-Saharan Africa due to SF antimalarials make up approximately 2.1 to 4.9% of total malaria deaths; and approximately 3.8 to 8.9% of malaria deaths relating to cases seeking treatment [5]. The threat has both public health and socioeconomic dimensions, as summarised in Fig. 1.

The WHO Global Surveillance and Monitoring System for substandard and falsified medicines, vaccines and in vitro diagnostics, based on data gathered in its first 4 years of operation, highlights the possible impact of SFs on individuals, families, national health systems and the economy and presents actual data from the surveillance system and other sources [6]. The report also describes the factors such as stock-outs, weak regulatory systems, absence of legal deterrents and weak enforcement that drives trade in SFs. It also discusses the systems and actions needed to prevent, detect and respond to the threats posed by SF medical products.

The risk posed by SF medical products is very real in the African region, which is currently leading in terms of the number of reports to the WHO rapid alert system. Forty-two percent of all reports to the WHO Global Surveillance and Monitoring System on SF medical products reported between 2013 and 2017 came from Africa, highlighting the magnitude of the problem [6]. However, the magnitude of the problem in the SADC region is not known.

A 2009 WHO baseline survey provided a general overview of the pharmaceutical situation in terms of demographic and economic aspects, health services data, medicines policy and access to medicines, including procurement and supply, intellectual property aspects and medicine regulation in the SADC region [7]. Information relating to the presence of SF products and related outcomes in the SADC region is also available in the public domain. WHO Medical Alert No 4/2015 confirmed circulation in the Democratic Republic of Congo (DRC) of falsified Diazepam tablets that caused acute dystonic reactions due to the presence of Haloperidol instead of Diazepam as the active ingredient. A survey conducted in 2014 to determine the quality of paediatric medicines sold by wholesalers in the Democratic Republic of Congo (DRC) revealed that 27.2% of samples collected were of poor quality; whilst 59.5% of collected artemether-lumefantrine samples had less than the required amount of artemether [8]. The same study report included findings from other surveys conducted in sub-Saharan Africa that indicated the prevalence of substandard antimalarials at the rate ranging from 12% in Tanzania to 88.4% in Malawi. Angola reported falsified artemether-lumefantrine and albendazole tablets in 2012, both medicines containing no active ingredient in them [9]. In a meta-analysis of studies conducted between 2009 and 2015, looking at the quality of artemisinin-based combination therapy products, the prevalence in Zambia of non-conforming products was 8% whilst a decrease in prevalence to 1.5% in 2015 from 25.5% in 2010 was observed for Madagascar. In this report, the prevalence in Tanzania was 7% [10]. In the period from July 2019 to June 2020 WHO issued 5 alerts targeting sub-Saharan Africa. They warned about the following products: falsified Augmentin found in Uganda and Kenya - August 2019, falsified Quinine Bisulphate circulating in Uganda and Quinine Sulphate circulating in Central African Republic and Chad - October 2019, falsified antimalarials in West and Central Africa displaying an outdated WHO Essential Drugs Programme logo – March 2020, falsified HIV rapid diagnostic test circulating in the WHO regions of the Americas and Africa – March-April 2020, falsified chloroquine products circulating in the WHO region of Africa - April and June 2020. Nonetheless [11], Despite the fact that the phenomenon itself is well documented, there is a scarcity of information with respect to the efforts in place to fight SF products. This article is a first step towards bridging this knowledge gap. 

The PDR approach recommended by WHO guides countries in protecting their health systems from the detrimental impact of substandard and falsified products. Their implementation requires engagement of a wide range of national stakeholders and cross-border collaborations. In August 2016, the Regional Committee for Africa at its sixty-sixth session adopted the Regional Strategy for Regulation of Medical Products, 2016–2025. Addressing the burden of substandard and falsified medical products was highlighted as one of the key elements for the African region in the strategy. The present survey was undertaken to monitor the progress in the implementation of the regional targets in the area of SF counteraction.

### Aim of the study

The study was conducted as part of an exercise to map the national approaches to counteract SF medical products in the WHO AFRO region. This article analyses the data describing national mechanisms to prevent, detect or respond to the incidence of SF medical products in the SADC region. The 2017 WHO GSN study report on SF medical products makes a case for collecting evidence. Strong evidence is needed to help prevent, detect and respond to SF products and the threat they represent. Therefore, the aim of the survey was to gather information that could form a basis to describe the status of national systems for counteracting SF medical products in the SADC region.

## Methodology

A survey was conducted between September 2018 and January 2019 to map the capacity and approaches for Prevention, Detection and Response to the incidence of substandard and falsified medical products by the National Medicines Regulatory Authorities (NMRAs) in the 16 Member States within SADC.

The survey was conducted using a structured, self-administered questionnaire developed in English, French and Portuguese. The development of the questionnaire was guided by the ‘Prevention, Detection and Response’ approach captured by the WHO Global Surveillance Network and approved by the Member State Mechanism; questions were structured around four themes, namely the presence of policies and frameworks, mandate and legislation, and coordination and response.

The tool underwent internal review in WHO and was amended upon the receipt of feedback from the inhouse experts.

The survey tool was distributed to the National Medicines Regulatory Authority (NMRA), which is either an autonomous or semi-autonomous regulatory authority or a department within the ministry of health, in each country. Return email addresses were provided on the questionnaire.

### Data collection and analysis

Results were received back from the NMRAs and compiled for use as part of the WHO mandate to generate evidence for effective health interventions and bring people to enjoy the highest attainable level of health and well-being. Obtained results were compiled on an Excel spreadsheet. For the purposes of data validation, the results were presented to the SADC NMRA heads present and the findings discussed at the 6th African Medicines Regulators’ Conference, which took place from 02–04 October 2019 in Victoria Falls, Zimbabwe.

## Results

The target group of the survey were NMRAs in the sixteen SADC countries. Twelve^2^ out of the sixteen countries responded to the survey translating it to a 75% response rate.

As the pool of respondents included all the countries in the SADC region and this being a small number, results are presented as absolute numbers and not in percentages. The survey results are outlined below and grouped into six thematic areas.

### Policy and implementation plan

**Fig. 2:**
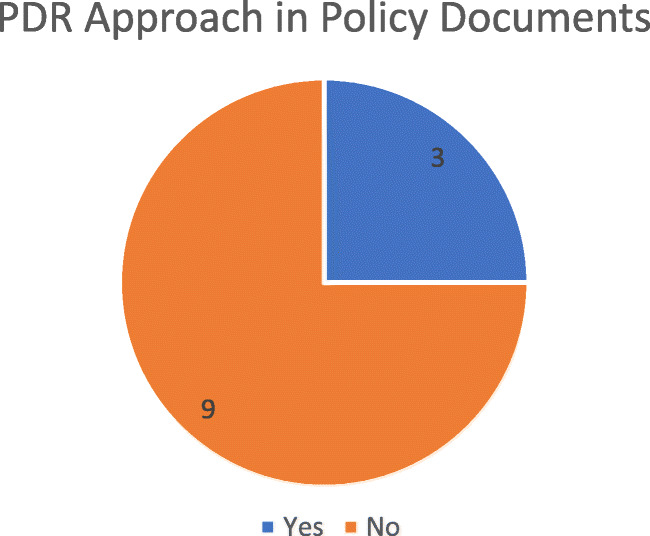
SF PDR approach included in policy documents

The number of NMRAs within SADC which had included elements for PDR to SF within their national medicines policies or strategy documents was very low, as shown in Fig. 2.

Most indicated that they had no policy frameworks to underpin prevention, detection or response to SF. Only three of the twelve respondent countries had this framework in place, with the same 3 being the only ones with implementation plans of action. It was noted that only one respondent that had a policy on SF had gone further and aligned their policies to the African Regional Strategy for Regulation of Medical Products, 2016–2025 endorsed by the 66th session of the WHO Regional Committee for Africa.

### Mandate and legislation

#### Criminal liability and sanctions

Nine out of twelve respondents indicated that they criminalised the manufacture, supply or distribution of SF products. Financial penalties and imprisonment were the most common sanctions provided for in national legislations. Jail terms ranging from not less than 6 months up to 15 years can be imposed. Out of the twelve respondents, five had legal provisions for seizure and confiscation of property other than the offending SF products.

### Administrative liability or sanctions

In the case of administrative liability or sanctions, they are defined as seizure and confiscation of SF products, suspension of the right to discharge official or professional functions, ban to practice or hold administrative or managerial positions and financial penalties. It was noted that they were applicable in nine of the twelve respondents. Of interest was that two of the responding NMRAs specified that they had provisions for criminal liability or administrative liability, and not both.

### Detection through a sampling of medical products

**Fig. 3:**
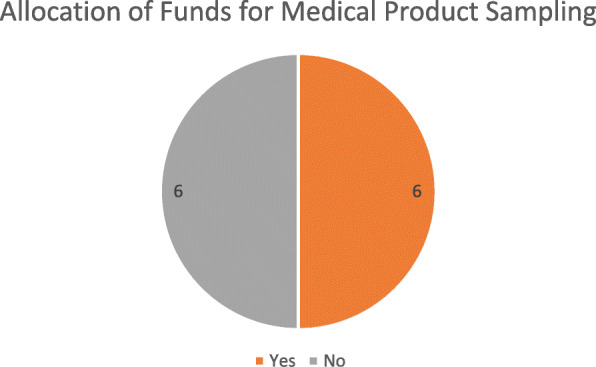
Allocation of funds for medical product sampling

The mandate for regular sampling was enshrined in the legislation of nine of the twelve respondent countries. As illustrated in Fig. 3, funds for implementation were allocated only in six.

A risk-based approach to sampling was applied by five of the respondents. Sampling plans were indicated to be regularly updated in six of the respondents that have a mandate to sample. The testing of the samples is carried out to some degree in the region. However, only three of the laboratories used were either ISO-certified or WHO-prequalified.

### Other sampling-related procedures

The survey captured information on other activities that are related to sampling. Half of the respondents, that is, six confirmed that documented procedures were in place to allow the implementation of decisions based on sample testing outcomes. Where SF products are detected, seven of the responding NMRAs had the authority and power to seize and dispose of them without having to resort to law enforcement agencies. However, only five of the respondents had the required infrastructure and documented procedures to ensure timely collection, handling, storage and in appropriate cases, disposal of samples or seized medical products that are unfit for use. Notably, one NMRA had the infrastructure and documented procedures for the handling of SF medical products but is legally required to involve law enforcers.

### Coordination and response mechanisms

Alleviating the SF burden requires engagement of all stakeholders and existence of coordination mechanisms. From the NMRA perspective, there are two types of stakeholders—internal and external. Internal stakeholders are NMRA departments, units and services responsible for various regulatory functions. External stakeholders include other health sector players, i.e., public health programmes, medical product procurement agencies, health professionals and their associations; representatives of other government authorities, such as law enforcers, investigators, judiciary and customs; and general public including mass media and patient communities. NMRAs were requested to indicate the existence of national interagency or interdisciplinary collaborative mechanisms such as national taskforces as to well as explain, which agencies were represented in these taskforces. Six respondents had both inter-agency and intra-agency co-ordination for SF products. They provided collaboration platforms for involving stakeholders to ensure coordination of the fight against SF trafficants. In six countries, there were also linkages between nationally coordinated mechanisms and national healthcare programmes.

### Surveillance of SF medical products

WHO has a global surveillance programme which produces medical product alerts to timely inform the healthcare stakeholders of the confirmed SF cases. The survey looked into the impact of this alert programme at the national health system level. Eleven of the twelve respondents reported regularly receiving the WHO alerts for SF medical products. Eight also reported having standard operating procedures to take action on the basis of the WHO alerts for SF medical products. One NMRA indicated that though a formal, written procedure was not in place, there were structured mechanisms to immediately alert professionals. Whilst the procedures were in place in almost all the NMRAs, the focus areas differed somewhat. All responding NMRAs that had procedures in place focussed on cascading the information to and on actions involving health professionals. Five of these also focussed on disseminating information to the general public, the extent to which this was done varying amongst the respondents.

## Discussion

The study was the first attempt to map national frameworks established for implementation of priority interventions for preventing, detecting and addressing substandard and falsified medical products in the SADC region.

There are two major streams of work in combating SF products. The first is the judicial approach, which is the preserve of the law enforcement agencies such as the police or the office of drug and crime. The focus in this approach is to introduce liability and in so doing, dissuade people from dealing with SF medical products. The second approach, taken by WHO and the focus of this article, puts forward the public health perspective to combine it with the judicial measures. SF medical products are a threat to public health, and NMRAs are the focal point for protecting public health with regard to medical products. In that regard, the survey targeted the NMRAs and looked in detail at their role in the fight against SF.

The study responses are the result of the review undertaken by the NMRAs with the help of the questionnaire. At present, there is no consensus on a global tool for assessing national systems and approaches to PDR for SF medical products. The survey could be the first step towards developing a substantive tool for evaluating PDR for SF products.

The study shows that policies and implementation plans are lacking for the majority of NMRAs. This largely affects guidance and direction in terms of the approach and strategy for the PDR for SF medical products, leaving the SADC region susceptible to such products being in their market. Notably, some NMRAs did include comments indicating that the need for formalising PDR policies had been identified in their strategic planning but was yet to be translated into policy documents and implementation plans.

Regionally, there is strength in terms of legislation. Nevertheless, limiting the penalties and sanctions to the seizure of detected samples or non-conforming medical products does not completely deal with the ill-gained wealth from criminal activities associated with their trafficking. Similarly, those NMRAs countries that do not include administrative liability or sanctions in their legislation must be encouraged to do so in order to put in place significant deterrents to engaging in trade in SF medical products.

Monitoring the supply chain, particularly through sampling and testing of products, is one of the measures for detecting SF products. A limitation noted was that there was a slew of countries without regular funding allocated collection of product samples for quality control testing which could affect the number of SF detection efficiency. At the time of the study, only three NMRAs had access to ISO-certified or WHO-prequalified laboratories. This has a negative impact on the confidence in and credibility of test results from the part of other NMRAs.

No NMRA has limitless resources for testing. In any case, it is not always feasible to test all products on the market. By the same token, there are significant costs associated with handling and storing detected SF products. This indicates the need to embrace the risk-based approach to sampling. NMRAs should also be encouraged to regularly update their sampling plans based on risk assessments in order to focus on susceptible products and have a relevant post-marketing surveillance system.

The trade in SF medical products is quite complex. Multi-pronged strategies are needed. Intra- and inter-agency co-ordination for responding to SF products, which was in existence in 50% of the countries, should be further strengthened and taken up by the other countries. A coordinated approach provides a platform for involving key stakeholders to ensure effectiveness of SF counteraction interventions.

The ability for NMRAs to seize and dispose of SF medical products without involving law enforcers balances out stakeholder involvement and allows medicine regulators to act swiftly, should the need arise. Issues of bureaucracy when involving other agencies could pose a risk for NMRAs without this mandate and could hamper the efficiency in responding to SF medical products.

Testing is essentially a means to an end. The true endpoint is enforcement of sound regulatory decisions based on the results of testing. NMRAs need to be assisted to utilise test results, starting with documenting procedures to do so.

Cognisance of and contributing to the WHO Global SF alerts, confirmed in eleven of the twelve respondents, are commendable. Equally commendable was the relatively high number of respondents who translated this to action, by putting in place putting in place and abiding by standard operating procedures. The study results did however give an indication of the need to broaden the scope and focus of actions. Public awareness and knowledge are powerful weapons in combating SF medical products, especially given the increasing complexity of global supply chains and the spread of e-commerce.

The study was an initial step in documenting the evidence of existing SF-counteraction frameworks in the SADC region of Africa. A limitation of the study lies in the methodology—a self-administered tool without external validation of the presented information. This calls for further efforts for comprehensive development of tools and devising acceptable mechanisms for data validation.

Constrained access to affordable, safe and quality medical products can lead to the proliferation of SF medical products [6, 12]. It is acknowledged that the access component was not included in the survey. The survey wanted to capture the perspective of the NMRAs, and they, by definition, are primarily concerned with quality, safety and efficacy of medical products. Though they support access, they may not necessarily be the best source of information about it. Access considerations are more from the demand side.

### Recommendations

There is a need to support NMRAs in the SADC region to strengthen PDR frameworks Access to ISO-certified or WHO prequalified testing facilities has to be increased.

Detection activities need to be further strengthened to stem the prevalence and to prevent clinical use of SF products. NMRAs may require training in risk-based post-marketing surveillance and practical implementation of its approaches. Ultimately, the national and regional commitment to the fight against SF medical products needs to be stepped up to ensure allocation of resources for implementation of prevention, detection and response activities in this vital area.

The inter-agency coordination to tackle SF medical products needs to be extended across borders, which are admittedly porous to SF medical products.

## Conclusion

Prevention, Detection and Response to SF products were in the SADC region call for further investments for strengthening existing frameworks. The availability of policies to guide intervention was low, and allocation of requisite funding to support the detection and testing of such products should be made a priority in national and regional agendas.

## Data Availability

The datasets used and analysed during the study that is the main basis for the review are available from the corresponding author on reasonable request.

## Abbreviations

DRC: Democratic Republic of Congo
GSN: Global Surveillance Network
NMRAs: National Medicines Regulatory Authorities
PDR: Prevention, Detection and Response
SADC: Southern African Development Community
SF: Substandard and falsified
WHO: World Health Organization

## Footnotes

1 *Substandard medical products*, also called ‘out of specification’ refers to authorised medical products that fail to meet either their quality standards, or their specifications or both. *Falsified medical products* are products that deliberately or fraudulently misrepresent their identity, composition or source.

2 Botswana, Comoros, Democratic Republic of Congo, Eswatini, Lesotho, Madagascar, Malawi, Mauritius, Mozambique, Namibia, Seychelles and Tanzania.
